# High SARS-CoV-2 seroprevalence in persons experiencing homelessness and shelter workers from a day-shelter in São Paulo, Brazil

**DOI:** 10.1371/journal.pntd.0009754

**Published:** 2021-10-19

**Authors:** Anahi Chechia do Couto, Louise Bach Kmetiuk, Ruana Renostro Delai, Ana Pérola Drulla Brandão, Cairo Oliveira Monteiro, Luciana Helena Antoniassi da Silva, Camila Soares, Alexandre Campos Banari, Renato van Wilpe Bach, Christina Pettan-Brewer, Andrea Pires dos Santos, Ana Marcia Sá Guimarães, Danielle Bruna Leal Oliveira, Edison Luiz Durigon, Alexander Welker Biondo

**Affiliations:** 1 Graduate College of Cellular and Molecular Biology, Federal University of Paraná (UFPR), Curitiba, Paraná, Brazil; 2 Department of Preventive Medicine, University of São Paulo Medical School (FMUSP), São Paulo, São Paulo, Brazil; 3 Department of Microbiology, Institute of Biomedical Sciences, University of São Paulo, São Paulo, Brazil; 4 Department of Medicine, State University of Ponta Grossa, Ponta Grossa, Paraná, Brazil; 5 Department of Comparative Medicine, University of Washington, Seattle, Washington, United States of America; 6 Department of Comparative Pathobiology, College of Veterinary Medicine, Purdue University, West Lafayette, Indiana, United States of America; Centre hospitalier de Cayenne, FRANCE

## Abstract

Brazil presents one of the highest COVID-19 death tolls in the world. The initial SARS-CoV-2 epicenter was São Paulo city. As of 2019, the homeless population of São Paulo city was estimated at 24,344 individuals, the largest national homeless population. The present study aimed to concomitantly assess the molecular and serological prevalence and associated risk factors of SARS-CoV-2 infection in a homeless population and related shelter workers from a day-shelter. Serum samples, nasopharyngeal and oropharyngeal swabs of persons who are homeless and shelter workers collected from August 25^th^ to 27^th^, 2020 were tested for the presence of anti-SARS-CoV-2 IgM and IgG antibodies by ELISA and SARS-CoV-2 RNA by RT-qPCR, respectively. All swab samples tested negative by RT-qPCR. Seropositivity of IgM and IgG was 5/203 (2.5%) and 111/203 (54.7%) in persons who are homeless, and 5/87 (5.7%) and 41/87 (47.1%) in shelter workers, respectively, with no statistical differences between groups. The high seroprevalence found herein indicates early environmental and urban spreading of SARS-CoV-2, associated with sociodemographic and economic vulnerability.

## Author summary

Brazil is one of the world’s most social-economically unequal countries, with a rising homeless population potentialized by the SARS-CoV-2 pandemic, particularly in its largest city, São Paulo. While few studies, mostly in high-income countries, have addressed the impact of the coronavirus pandemic on homeless populations, none has been carried out in Brazil. Herein, we report a high SARS-CoV-2 IgG seroprevalence in a homeless population, with 111/203 (54.7%) seropositive individuals. At the time of the study (August 25^th^- 27^th^ 2020), both homeless and social worker populations showed no active SARS-CoV-2 infection, indicating that they were likely exposed sometime within the pandemic’s first peak in the city. Our study has also shown significant risk and protective factors for SARS-CoV-2 infection, including that Black shelter workers were at higher risk of SARS-CoV-2 infection when compared with the white shelter workers.

## Introduction

The current SARS-CoV-2 pandemic has severely affected Latin America, particularly Brazil, currently presenting one of the highest active transmission rates among 48 countries, with 16,720,081 confirmed cases and 467,706 deaths as of June 1^st^, 2021 [1]. Due to pre-existing socioeconomic inequalities, the novel coronavirus spread has affected vulnerable populations worldwide and impacted human social welfare [2]. The pandemics has also increased their vulnerability as a consequence of social and economic losses, associated with disparities in policy responses, particularly in emerging countries [3].

Brazil has been ranked as the largest and most unequal Latin American country in income distribution (Gini index of 0.540 in 2018) [4], with inequality rising since 2014 as a result of economic crisis and political turmoil, with 13.6 million people living in extreme poverty, 6.5% of the overall nationwide population [4]. Aggravated by the SARS-CoV-2 pandemic, extreme poverty in Brazil has been expected to rise 9.5% by the end of 2020 [5], leading to an increase in homelessness, particularly in major urban centers. Since 2012, the homeless population in Brazil has grown around 140%, reaching almost 222,000 people in 2019, with more than half (56.2%) living in south-eastern Brazil, mainly in Sao Paulo city [6]. As the most populous Brazilian city and the fourth worldwide, São Paulo had an estimated homeless population around 24,000 persons in 2019. In addition to insufficient healthcare access, inadequate nutrition, and inability to prevent SARS-CoV-2 transmission due to precarious living conditions [3], such population has presented multiple comorbidities, such as drug addiction, sexually transmitted and other infectious diseases, and non-communicable diseases, with some being associated with worsening the clinical onset of SARS-CoV-2 infection [7,8].

Few studies have been conducted on SARS-CoV-2 detection in persons experiencing homelessness or shelter workers, mostly taken at the beginning of local epidemics between March and April 2020. In the USA, the prevalence of SARS-CoV-2 by RT-qPCR in cohabitants and support service workers (shelter workers) of various institutions varied from 48/533 (9.0%) in Seattle, and 162/458 (35.4%) and 147/408 (36.0%) in Boston, 105/206 (50.9%) in San Francisco, 11/308 (3.6%) in Atlanta, and 18/118 (15.2%) and 19/181 (10.5%) in King County, Washington State [9,10]. No survey to date has been conducted in Brazilian homeless populations. Thus, this study aimed to concomitantly assess the molecular and serological prevalence and associated risk factors of SARS-CoV-2 infection in a homeless population and shelter workers from a day-shelter in São Paulo City, the urban epicenter of SARS-CoV-2 transmission in Brazil at the time of the survey.

## Material and methods

### Local of study

This is a cross-sectional study of a homeless population and related shelter workers. These shelter workers were healthcare and assistance professionals (e.g., nurses, social workers, administrative personnel, cooks, cleaning, and maintenance professionals) providing care to people who were homeless. The study was conducted in the city of São Paulo (23°33’1"S, 46°38’2"W), capital of São Paulo State, south-eastern Brazil, ranked as the second largest Gross Domestic Product (GDP) and the most populated city in Latin America, with 11,253,500 habitants, a high Human Development Index (HDI) (0.805), humid subtropical climate and average temperatures varying from 19°C (winter) to 25°C (summer) [11].

**Fig 1:**
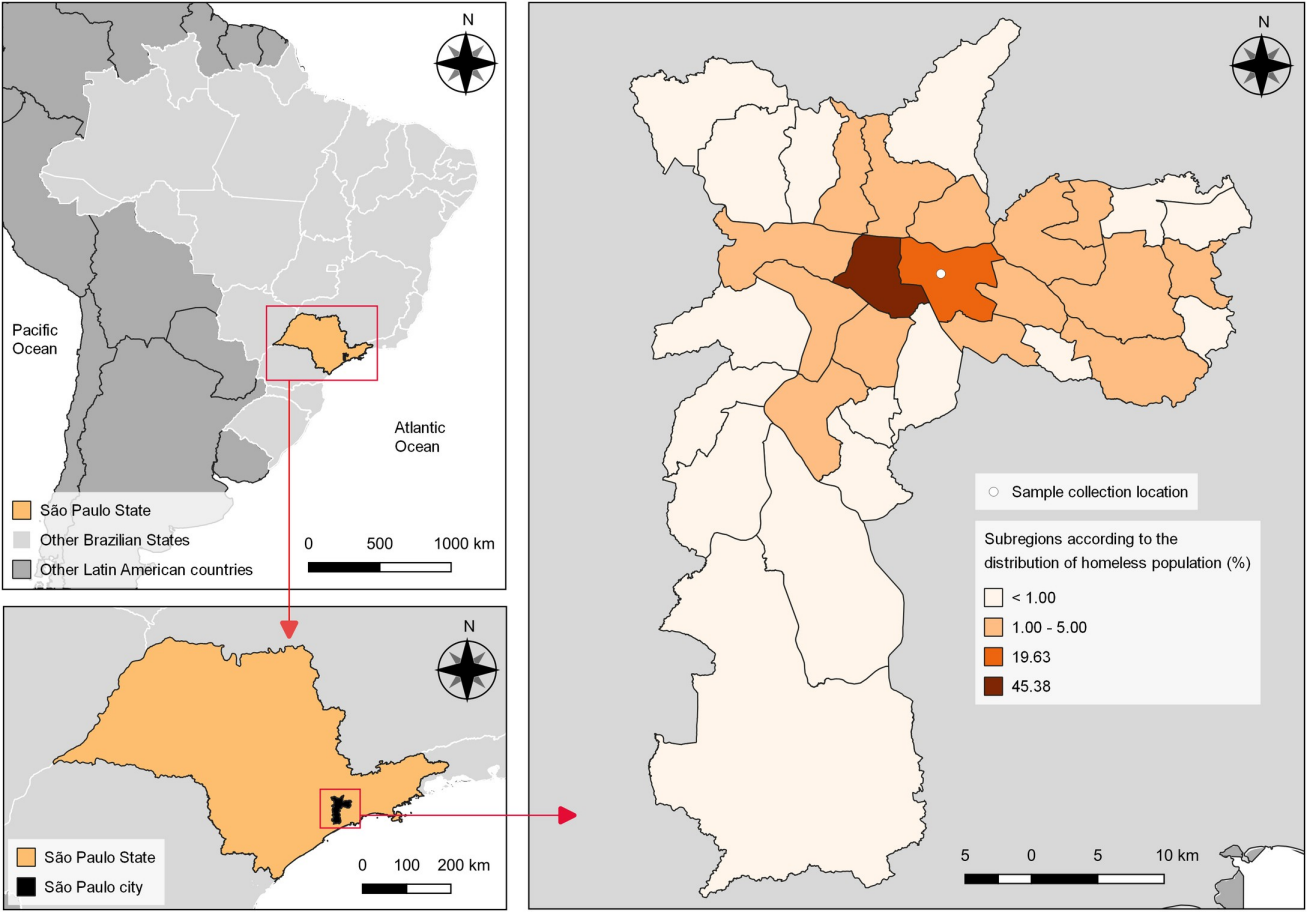
Geographical location of the shelter and distribution of homeless population as described in the latest São Paulo city survey [12]. All maps are public domain (https://www.ibge.gov.br/geociencias/downloads-geociencias.html).

Sample collection was performed in the consecutive working days of August 25^th^, 26^th,^ and 27^th^, 2020, at a major shelter for people who are homeless called the Community Center of São Martinho de Lima, located in the Mooca subregion, area with the second highest homeless population, with 4,779 individuals corresponding to 19.6% of the total homeless population (*n* = 24,344) of São Paulo City [12] (Fig 1). The center is a day-only public service with no dormitory or sleepover, providing three daily meals and medical assistance to persons who are homeless. The shelter serves around 600 breakfast meals and 800 lunch meals daily. The exact number of persons experiencing homelessness accessing the center each day is unknown. In a scenario where 800 people access the shelter daily (i.e., the maximum capacity for lunch meals), we calculated a sample size of 204 people for this study (50% SARS-CoV-2 prevalence, 90% CI, and 5% error). In addition, the shelter has around 90 workers that assist with cleaning, cooking, and maintenance, in addition to social and healthcare professionals.

Individuals who are homeless were informed about the COVID-19 testing research by public announcement as they entered the shelter. The reason why these individuals were entering the shelter was not asked and they voluntarily entered the sampling line. As no individual invitation was made for participation, no calculation of refusal rate was possible. The interviewers and medical team were stationed inside the first and largest room of the center and worked every day from 7 am (before breakfast) to around 3 pm (after lunch). Meanwhile, shelter workers were informed about the COVID-19 testing research two days prior to sampling. They also voluntarily came to sampling lines and were received by the interviewers and medical team. All shelter workers from the center participated in the study. Participants were first informed about the study, provided signed consent, responded to the questionnaire, and then were subjected to blood and swab collection. To be included in the study, participants needed to have answered the questionnaire and collected both blood and swab samples.

This study has been approved by the Ethics Committee in Research at the Federal University of Paraná (CAAE: 80099017.3.0000.0102, protocol number: 2.512.196), by the Municipal Ethics in Health Committee, São Paulo Secretary of Health (CAAE: 80099017.3.3004.0086, protocol number: 3.366.684) and by the Ethics Committee Research of the Clinical Hospital from the Federal University of Paraná (CAAE: 80099017.3.3005.0096, protocol number: 3.623.845), linked to the National Human Ethics Research Committee of the Brazilian Ministry of Health. The investigation was carried out in coordination with the shelter service providers, healthcare providers, and universities. All participants (who were all > 18 years old) were informed and signed a written consent.

### Application of a structured epidemiological questionnaire

All participants (both persons who are homeless and shelter workers) were interviewed with a structured questionnaire for sociodemographic, behavioral, and clinical information. For the homeless population, questions evaluated sociodemographic aspects (city of origin, age, gender, self-identified race/ethnicity, and level of education), previous assistance by the government’s healthcare program “Street Clinics” (“Consultório na Rua”), previous assistance by counselling and psychological services (CAPS), current drug use (alcohol, tobacco, marijuana, cocaine, crack, injectables–yes or no questions), number of years without permanent housing, reasons for homelessness (unemployment, alcohol/drugs, familiar conflict, others), use of face mask, previous contact with someone positive for SARS-CoV-2, current or previous (last three months) SARS-CoV-2 symptoms (fever, difficulty breathing, tiredness, body pain or discomfort, throat pain, diarrhea, chest pain, dry cough, loss of sense of smell or taste, headache), previous SARS-CoV-2 testing, ownership of companion animal(s) (dog, cat, other), previous health history (HIV, syphilis, hepatitis, cardiovascular disease, tuberculosis, diabetes, other comorbidities), and access to water and soap to wash hands.

For shelter workers, questions evaluated sociodemographic aspects (city of origin, age, gender, self-identified race/ethnicity, level of education), current drug use (tobacco, marijuana, cocaine, crack, injectables–yes or no questions), use of face mask, previous contact with someone positive for SARS-CoV-2, current or previous (last three months) SARS-CoV-2 symptoms (fever, difficulty breathing, tiredness, body pain or discomfort, throat pain, diarrhea, chest pain, dry cough, loss of sense of smell or taste, headache), previous SARS-CoV-2 testing, ownership of companion animal (dog, cat, other), previous health history (HIV, syphilis, hepatitis, cardiovascular disease, tuberculosis, diabetes, other comorbidities), and access to water and soap to wash hands. All answers provided by the participants (individuals who are homeless or shelter workers), including symptoms and comorbidities, were self-reported.

### Sample collection

Nasopharyngeal and oropharyngeal swabs and whole blood samples were collected from individuals by trained nurses and subjected to SARS-CoV-2-specific RT-qPCR and ELISA (IgM and IgG) assays, respectively. Briefly, nasopharyngeal and oropharyngeal swabs from each patient were jointly stored in a cryotube containing 1 mL of lysis buffer (NucliSENS easyMag, BioMerieux, Lyon, France) for virus inactivation and preservation. Blood samples and cryotubes containing the swabs were refrigerated and processed on the same day at the Institute of Biomedical Sciences (ICB), University of São Paulo (USP), Brazil. The serum was separated after centrifugation of the whole blood samples at 2,500 x g for 10 minutes and stored at 4°C until testing.

### SARS-CoV-2 RT-qPCR

Nasopharyngeal and oropharyngeal swabs were processed in the Laboratory of Clinical and Molecular Virology (LVCM), ICB, USP for molecular SARS-CoV-2 testing by a specific RT-qPCR [13]. First, total RNA was extracted from 400 μL of sample using NucliSENS easyMag fully automated platform (BioMerieux, Lyon, France). The RT-qPCR assay was then carried out using an adapted protocol developed at the Charité Institute of Virology, University of Berlin, Germany [13]. The positive control consisted of RNA extracted from Vero-E6 cell culture infected with SARS-CoV-2 (SARS.COV-2/SP02/human2020/Br, GenBank accession number MT126808.1), and the negative control was ultrapure water. In addition, RT-qPCR for the housekeeping gene human RNase P (RNP) was run to ensure RNA integrity, sample quality, and absence of inhibitors, as described previously [14]. All samples were run in duplicates.

### Serological Test

IgM and IgG antibodies against the SARS-CoV-2 nucleocapsid (N) protein were measured using a previously developed ELISA assay [15]. Briefly, 96-well polystyrene microliter plates (Corning, NY, USA) were coated with 100 μL of Ncov-PS-Ag7 antigen (Fapon Biotech Inc, Dongguan, China) at a concentration of 0.2 μg/mL in 0.05 M sodium carbonate buffer (pH 9.6) for one hour at 37°C. The plates were then washed with 1X phosphate-buffered saline with 0.05% Tween 20 (PBST) five times and blocked with 300 μL/well of blocking buffer (Advagen Biotech®, São Paulo, Brazil) for 3 hours at 37°C. A total of 10 μL of each serum sample was diluted at 1:50 for IgM, and 1:100 for IgG in diluent solution added to each well and incubated for one hour at 37°C. Following five washes with PBST, bound antibodies were detected using goat anti-human IgM (1:4,000) or IgG (1:4,000) conjugated with horseradish peroxidase (Sigma-Aldrich Co., Steinheim, Germany). Immunoglobulin detection was revealed after five washes with PBST and 10 minutes incubation with tetramethylbenzidine (Invitrogen, California, USA) at room temperature. After stopping the reaction with 0.2 N sulfuric acid, the optical density (O.D.) was measured at 450 nm. Two positive serum samples and three negative serum samples were used as controls. The two positive serum samples were from symptomatic patients confirmed to be infected with SARS-CoV-2 by RT-qPCR, while the negative serum samples were from the pre-pandemic period. For IgM, the cut-off value was determined using the average O.D. of the three negative serum samples plus three standard deviations, while for IgG was set as 0.4, as previously described [15].

### Data collection and statistical analysis

To identify the pandemic’s epidemiological moment when the survey was carried out, data regarding reported cases and deaths from March 28^th^ (first case of SARS-CoV-2 detected in Brazil) to November 19^th^, 2020 in São Paulo city were retrieved from the official records of the Brazilian Ministry of Health (https://susanalitico.saude.gov.br/extensions/covid-19_html/covid-19_html.html) and plotted against time using Microsoft Excel 365. Questionnaire data and serological test results were organized in spreadsheets and analyzed in R software, version 4.0.3 [16] to verify associations between studied variables and serology results for SARS-CoV-2 (IgG). The positive results between groups were compared with Pearson’s chi-square test. For each group, a bivariate analysis for all independent variables was performed by calculating the Odds Ratio (OR), the Confidence Interval (CI) for OR and the p-value, with a confidence level (α) of 5%. Then, a multivariate analysis was performed fitting variables in a logistic regression model (stepwise logistic regression). Using a forward stepwise approach and adjusting the models for age and sex, the best fitting model was the one including significantly associated variables (p ≤ 0.05) and minimizing the Akaike’s Information Criterion (AIC) value.

Constant variables—those that all respondents gave the same answer to–or collinear variables were excluded from the final model, as well as variables with more than 10% of missing data. These include: for the homeless population–years experiencing homelessness (missing values = 52), reasons for homelessness (unemployment, alcohol/drugs, familiar conflict, others) (missing values = 29), companion animal (other) (Constant = no); and for shelter workers—drug use (cocaine, crack, injectables) (Constant = no), selected current or previous SARS- CoV-2 symptoms (yes x no) (difficulty breathing, tiredness, throat pain, diarrhea, chest pain, dry cough) (Collinearity), previous health history (yes x no) (hepatitis, tuberculosis) (Constant = no).

Health histories, including infectious diseases, diabetes, and cardiovascular diseases, were compared between persons who are homeless and shelter workers using Pearson’s chi-square or Fisher exact tests. Optical density distributions of IgG titers were compared between populations using Mann-Whitney test. Results were considered significant when *p* ≤ 0.05.

## Results

A total of 203 individuals who are homeless and 87 shelter workers participated in the study. Twenty-nine additional individuals experiencing homelessness answered only the questionnaire but refused to be sampled, and other four refused to collect swab samples; these were all excluded from the study. The 290 swab and serum samples were submitted to SARS-CoV-2-specific RT-qPCR and ELISA testing. Successful amplification of housekeeping gene RNP control has indicated that all RNA samples were suitable for testing, with Ct values ranging from 23.1 to 29.9 (mean = 26.1, stdev = 1.4). None of the 290 RNA samples from swabs was positive in the SARS-CoV-2-specific RT-qPCR assay, indicating the absence of active infection in the surveyed populations.

**Fig 2:**
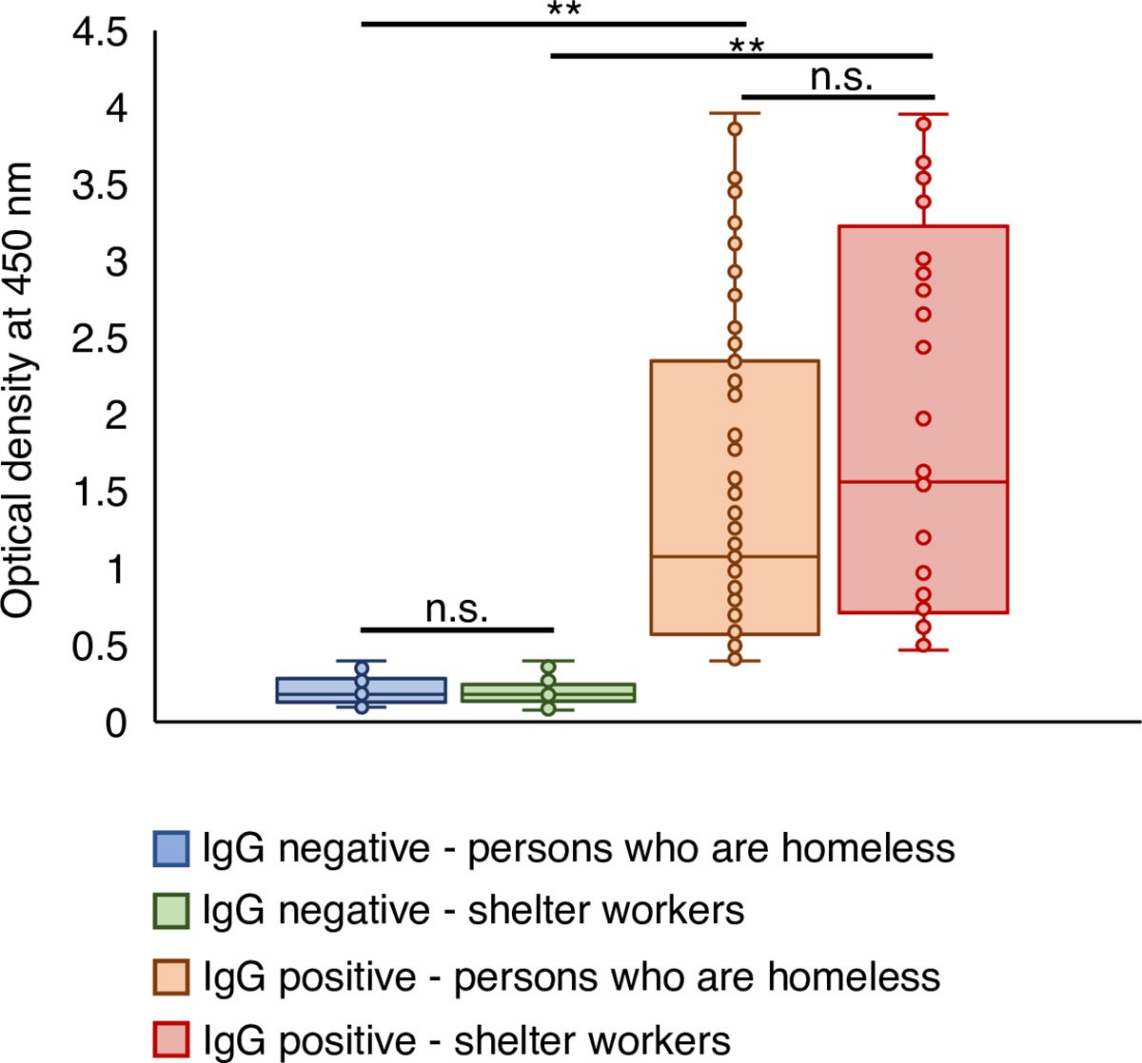
Optical density at 450 nm obtained for the ELISA assay used to detected anti-SARS-CoV-2 IgG in persons who are homeless (n = 203) and shelter workers (n = 87) attending the Community Center of São Martinho de Lima, Mooca subregion, São Paulo, Brazil. ELISA assay was performed as described in materials and methods. **p ≤ 0.05 (Positive IgG–individuals who are homeless versus shelter workers: p = 0.025), n.s.: not significant (Negative IgG–individuals who are homeless versus shelter workers: p = 0.901). Statistical analysis was performed using the Mann-Whitney test.

In contrast, IgM and IgG antibodies were found in 5/203 (2.5%, CI 0.3–4.6%) and 111/203 (54.7% CI 47.8–61.5%) persons who are homeless, and in 5/87 (5.7%, CI 0.9–10.6%) and 41/87 (47.1%, CI 36.6–57.6%) shelter workers, with no statistical differences between the two populational groups (IgM *p* = 0.291; IgG *p* = 0.251). Presence of IgM in the absence of IgG was not observed in the individuals who are homeless, while two (2.3%) shelter workers had circulating IgM and no IgG. Optical densities for the 10 IgM-positive individuals ranged from 0.431 to 1.967, while the distributions of the optical density values of IgG obtained are shown in Fig 2, with significant difference in ELISA titer being detected between individuals who are homeless and shelter workers (*p* = 0.025). These findings indicate that SARS-CoV-2 infection in these populations was not a recent event, corroborating with the negative results observed in the SARS-CoV-2 RT-qPCR. As very few individuals had IgM antibodies only, IgG seropositivity was considered the gold standard to indicate previous SARS-CoV-2 infection and was used in the risk factor analysis.

**Fig 3:**
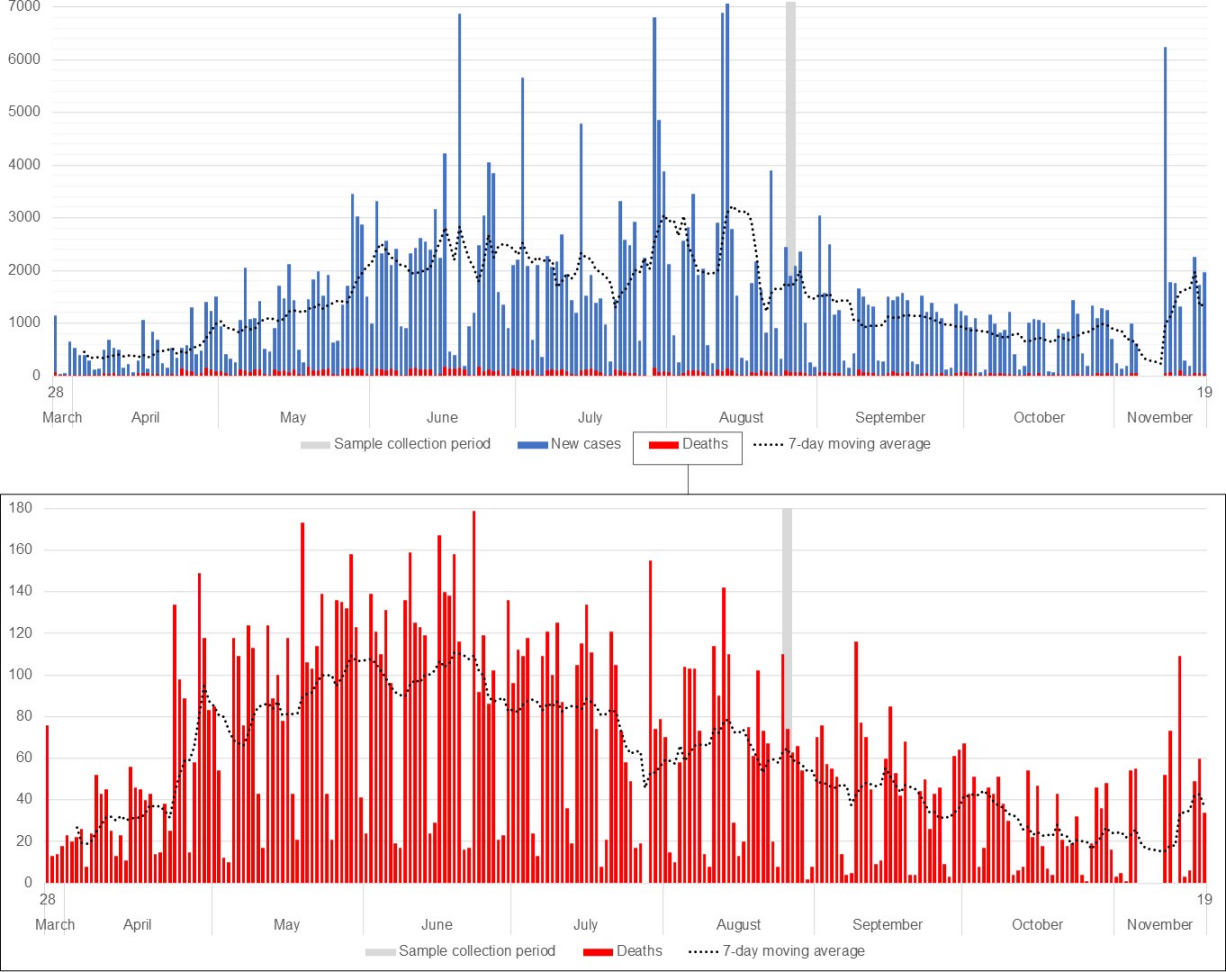
Number of SARS-CoV-2 new cases (upper graph, blue) and deaths (upper and bottom graphs, red) officially reported in São Paulo city, Brazil, from March 28^th^ (first case of SARS-CoV-2 detected in Brazil) to November 19^th,^ 2020, with a 7-day moving average. Days of sample collection are shown as grey bars (August 25^th^ to 27^th^, 2020).

Using official data from the Brazilian Ministry of Health of reported new cases and deaths of SARS-CoV-2 in São Paulo city, at the time of the survey, a decline in the numbers from its first initial peak (end of June 2020 for deaths) was observed, presenting a moving average from 1,706.86 to 1,768.86 new cases and from 59.29 to 64.86 deaths reported daily at that epidemiological week [17] (Fig 3). The containment phase was determined as orange (from a red to green scale, where green was medium risk with fewer restrictions, and red was very high risk with essential activities only) by São Paulo Health Authorities. Thus, at the time of the survey, the first highest peak of deaths by COVID-19 in the city had passed, although viral transmission was not controlled.

The descriptive statistics of the surveyed population is presented in Tables 1 and 2. Contrasting sociodemographic characteristics (except for age) and health histories were observed between homeless and shelter worker populations. While the majority of the shelter workers were born in São Paulo (61.2%), individuals who are homeless were mostly (68%) from other Brazilian cities, reflecting the well-described phenomena of migratory movements of vulnerable populations to large urban centers in the country [18]. Additionally, persons who are homeless were mostly male (89.1%), while the shelter worker population was well distributed between males and females. Furthermore, most individuals who are homeless identified themselves as Brown (mixed-race Brazilians) (42.9%), followed by white (29.5%), Black (26.1%), and Indigenous Brazilian (1.5%), while the shelter workers were somewhat equally represented by white (35.3%), Black (31.8%), and Brown (32.9%). Finally, 89.7% of the shelter workers had high school and/or higher education diplomas compared with 52.2% of the homeless population.

**Table 1:** Descriptive statistics and results of bivariate analysis of associated risk factors for seropositivity of anti-SARS-CoV-2 IgG in 203 persons who are homeless of São Paulo, Brazil.

	Variables	Descriptive statistics								Crude OR	95% CI		p-value
		Negative		Positive		Total		Answer rate					
		n	%	n	%	n	%	n	%				
Sociodemographic aspects	**Origin**												
	São Paulo	30	32.6	35	31.5	65	32.0	203	100.0	Ref			
	Others	62	67.4	76	68.5	138	68.0			1.05	0.58	1.90	0.870
	**Age** *												
	Young (18–30 years-old)	21	22.8	8	7.3	29	14.4	202	99.5	Ref			
	Adult (31–60 years old)	62	67.4	79	71.8	141	69.8			3.35	1.44	8.51	**0.007**
	Elderly (> 60 years old)	9	9.8	23	20.9	32	15.8			6.71	2.27	21.75	**< 0.001**
	**Gender**												
	Male	82	90.1	98	88.3	180	89.1	202	99.5	Ref			
	Female	8	8.8	12	10.8	20	9.9			1.26	0.50	3.34	0.636
	Others	1	1.1	1	0.9	2	1.0			0.84	0.03	21.38	0.900
	**Race/Ethnicity**												
	White	30	32.6	30	27.0	60	29.6	203	100.0	Ref			
	Black	20	21.7	33	29.7	53	26.1			1.65	0.78	3.50	0.191
	Mixed (Brown—Pardo)	41	44.6	46	41.4	87	42.9			1.12	0.58	2.17	0.732
	Indigenous Brazilian	1	1.1	2	1.8	3	1.5			2.00	0.17	23.25	0.580
	**Level of education**												
	Higher education	7	7.6	12	10.8	19	9.4	203	100.0	Ref			
	High school	46	50.0	41	36.9	87	42.9			0.52	0.19	1.45	0.210
	Elementary school	39	42.4	58	52.3	97	47.8			0.87	0.31	2.40	0.784
Previous assistance	**Assistance by the government healthcare program**												
	Yes	27	29.7	40	37.4	67	33.8	198	97.5	Ref			
	No	64	70.3	67	62.6	131	66.2			0.71	0.39	1.28	0.254
	**Assistance by Psychosocial Care Centers**												
	Yes	26	28.3	29	26.4	55	27.2	202	99.5	Ref			
	No	66	71.7	81	73.6	147	72.8			1.10	0.59	2.05	0.763
Behavior characteristics	**Alcohol**												
	No	34	37.0	46	41.4	80	39.4	203	100.0	Ref			
	Yes	58	63.0	65	58.6	123	60.6			0.83	0.47	1.46	0.515
	**Tobacco** *												
	No	31	33.7	59	53.2	90	44.3	203	100.0	Ref			
	Yes	61	66.3	52	46.8	113	55.7			0.45	0.25	0.79	**0.006**
	**Marijuana** *												
	No	55	59.8	88	79.3	143	70.4	203	100.0	Ref			
	Yes	37	40.2	23	20.7	60	29.6			0.39	0.21	0.72	**0.003**
	**Cocaine**												
	No	55	59.8	80	72.1	135	66.5	203	100.0	Ref			
	Yes	37	40.2	31	27.9	68	33.5			0.58	0.32	1.04	0.066
	**Crack**												
	No	69	75.0	87	78.4	156	76.8	203	100.0	Ref			
	Yes	23	25.0	24	21.6	47	23.2			0.83	0.43	1.60	0.570
	**Injectables**												
	No	84	93.3	111	100.0	195	97.0	201	99.0	Ref			
	Yes	6	6.7	0	0.0	6	3.0			0.06	0.00	1.05	0.054
Years experiencing homelessness	**Years on the streets**												
	Less than one year	23	33.3	28	34.1	51	33.8	151	74.4	Ref			
	One year or more	46	66.7	54	65.9	100	66.2			0.96	0.49	1.90	0.916
Reasons for homelessness	**Unemployment**												
	No	38	48.1	42	44.2	80	46.0	174	85.7	Ref			
	Yes	41	51.9	53	55.8	94	54.0			1.17	0.64	2.13	0.608
	**Alcohol/drugs**												
	No	53	67.1	69	72.6	122	70.1	174	85.7	Ref			
	Yes	26	32.9	26	27.4	52	29.9			0.77	0.40	1.47	0.427
	**Familiar conflict**												
	No	47	59.5	53	55.8	100	57.5	174	85.7	Ref			
	Yes	32	40.5	42	44.2	74	42.5			1.16	0.64	2.14	0.623
	**Other**												
	No	76	95.0	92	96.8	168	96.0	175	86.2	Ref			
	Yes	4	5.0	3	3.2	7	4.0			0.62	0.12	2.89	0.539
Use of face mask during pandemic	**Face mask use**												
	Yes	75	83.3	97	88.2	172	86.0	200	98.5	Ref			
	No	15	16.7	13	11.8	28	14.0			0.67	0.30	1.49	0.328
Contact history	**Contact with someone positive**												
	No	68	75.6	80	73.4	148	74.4	199	98.0	Ref			
	Yes	22	24.4	29	26.6	51	25.6			1.12	0.59	2.14	0.728
Current or previous (last 3 months) COVID-19 symptoms	**Fever**												
	No	74	80.4	80	72.1	154	75.9	203	100.0	Ref			
	Yes	18	19.6	31	27.9	49	24.1			1.59	0.83	3.13	0.168
	**Difficulty breathing**												
	No	67	72.8	76	68.5	143	70.4	203	100.0	Ref			
	Yes	25	27.2	35	31.5	60	29.6			1.23	0.67	2.29	0.499
	**Tiredness**												
	No	68	73.9	77	69.4	145	71.4	203	100.0	Ref			
	Yes	24	26.1	34	30.6	58	28.6			1.25	0.68	2.33	0.476
	**Body pain**												
	No	63	69.2	86	77.5	149	73.8	202	99.5	Ref			
	Yes	28	30.8	25	22.5	53	26.2			0.65	0.35	1.23	0.186
	**Throat pain**												
	No	74	80.4	86	77.5	160	78.8	203	100.0	Ref			
	Yes	18	19.6	25	22.5	43	21.2			1.20	0.61	2.39	0.608
	**Diarrhea**												
	No	77	83.7	97	87.4	174	85.7	203	100.0	Ref			
	Yes	15	16.3	14	12.6	29	14.3			0.74	0.33	1.63	0.455
	**Chest pain**												
	No	73	79.3	88	79.3	161	79.3	203	100.0	Ref			
	Yes	19	20.7	23	20.7	42	20.7			1.00	0.51	2.00	0.990
	**Dry cough**												
	No	63	68.5	86	77.5	149	73.4	203	100.0	Ref			
	Yes	29	31.5	25	22.5	54	26.6			0.63	0.34	1.18	0.150
	**Loss of sense of smell or taste**												
	No	77	83.7	90	81.1	167	82.3	203	100.0	Ref			
	Yes	15	16.3	21	18.9	36	17.7			1.20	0.58	2.52	0.628
	**Headache**												
	No	68	73.9	78	70.3	146	71.9	203	100.0	Ref			
	Yes	24	26.1	33	29.7	57	28.1			1.20	0.65	2.24	0.566
Previous COVID-19 test	**Previous COVID-19 test**												
	Yes	7	7.6	16	14.4	23	11.3	203	100.0	Ref			
	No	85	92.4	95	85.6	180	88.7			2.05	0.83	5.54	0.134
Companion animals	**Companion animal** *												
	No	71	77.2	101	91.0	172	84.7	203	100.0	Ref			
	Yes	21	22.8	10	9.0	31	15.3			0.33	0.14	0.74	**0.008**
	**Dog** *												
	No	78	84.8	104	93.7	182	89.7	203	100.0	Ref			
	Yes	14	15.2	7	6.3	21	10.3			0.38	0.14	0.95	**0.044**
	**Cat** *												
	No	83	90.2	108	97.3	191	94.1	203	100.0	Ref			
	Yes	9	9.8	3	2.7	12	5.9			0.26	0.06	0.89	**0.046**
	**Other**												
	No	92	100.0	111	100.0	203	100.0	203	100.0	Ref			
	Yes	0	0.0	0	0.0	0	0.0			0.83	0.02	42.22	0.926
Health history	**HIV**												
	No	83	92.2	104	94.5	187	93.5	200	98.5	Ref			
	Yes	7	7.8	6	5.5	13	6.5			0.68	0.21	2.13	0.509
	**Syphilis**												
	No	78	86.7	91	83.5	169	84.9	199	98.0	Ref			
	Yes	12	13.3	18	16.5	30	15.1			1.29	0.59	2.90	0.533
	**Hepatitis**												
	No	85	93.4	99	90.0	184	91.5	201	99.0	Ref			
	Yes	6	6.6	11	10.0	17	8.5			1.57	0.57	4.74	0.391
	**Cardiovascular diseases**												
	No	68	73.9	76	68.5	144	70.9	203	100.0	Ref			
	Yes	24	26.1	35	31.5	59	29.1			1.30	0.71	2.43	0.396
	**Tuberculosis**												
	No	81	88.0	97	89.0	178	88.6	201	99.0	Ref			
	Yes	11	12.0	12	11.0	23	11.4			0.91	0.38	2.20	0.834
	**Diabetes**												
	No	85	92.4	99	89.2	184	90.6	203	100.0	Ref			
	Yes	7	7.6	12	10.8	19	9.4			1.47	0.57	4.11	0.438
	**Other diseases**												
	No	78	85.7	94	85.5	172	85.6	201	99.0	Ref			
	Yes	13	14.3	16	14.5	29	14.4			1.02	0.46	2.25	0.958
Wash hands with water and soap	**Water and soap**												
	Yes	81	88.0	102	92.7	183	90.6	202	99.5	Ref			
	No	11	12.0	8	7.3	19	9.4			0.58	0.22	1.50	0.261

Ref = Category of reference

* Statistically significant variables

**Table 2:** Descriptive statistics and results of bivariate analysis of risk factors associated with seropositivity of anti-SARS-CoV-2 IgG in 87 shelter workers of São Paulo, Brazil.

	Variables	Descriptive statistics								Crude OR	95% CI		p-value
		Negative		Positive		Total		Answer rate					
		n	%	n	%	n	%	n	%				
Sociodemographic aspects	**Origin**												
	São Paulo	32	72.7	20	48.8	52	61.2	85	97.7	Ref			
	Others	12	27.3	21	51.2	33	38.8			2.80	1.15	7.07	**0.025**
	**Age**												
	Young (18–30 years-old)	9	19.6	6	14.6	15	17.2	87	97.7	Ref			
	Adult (31–60 years old)	35	76.1	34	83.0	69	79.3			1.46	0.47	4.76	0.516
	Elderly (> 60 years old)	2	4.3	1	2.4	3	3.5			0.75	0.03	9.68	0.829
	**Gender**												
	Male	24	52.2	20	48.8	44	50.6	87	100.0	Ref			
	Female	22	47.8	21	51.2	43	49.4			1.15	0.49	2.67	0.752
	**Race/Ethnicity**												
	White	21	47.7	9	21.9	30	35.3	85	97.7	Ref			
	Black	10	22.7	17	41.5	27	31.8			3.97	1.35	12.47	**0.015**
	Mixed (Pardo)	13	29.6	15	36.6	28	32.9			2.69	0.93	8.17	0.072
	**Level of education**												
	Higher education	25	54.3	16	39.0	41	47.1	87	97.7	Ref			
	High school	16	34.8	21	51.2	37	42.5			2.05	0.84	5.14	0.119
	Elementary school	5	10.9	4	9.8	9	10.4			1.25	0.27	5.43	0.764
	**Tabacco**												
	No	33	71.7	34	85.0	67	77.9	86	98.9	Ref			
	Yes	13	28.3	6	15.0	19	22.1			0.45	0.14	1.28	0.145
	**Marijuana**												
	No	45	97.83	39	97.50	84	97.67	86	98.9	Ref			
	Yes	1	2.17	1	2.50	2	2.33			1.15	0.04	29.83	0.920
	**Cocaine**												
	No	46	100.00	39	97.50	85	98.84	86	98.9	Ref			
	Yes	0	0.00	1	2.50	1	1.16			3.53	0.14	89.16	0.444
	**Crack**												
	No	46	100.00	40	100.00	86	100.00	86	98.9	Ref			
	Yes	0	0.00	0	0.00	0	0.00			1.24	0.63	2.40	0.527
	**Injetables**												
	No	46	100.00	40	100.00	86	100.00	86	98.9	Ref			
	Yes	0	0.00	0	0.00	0	0.00			0.78	0.11	4.08	0.773
Use of face mask during pandemic	**Face mask use**												
	Yes	45	97.83	39	97.50	84	97.67	86	98.9	Ref			
	No	1	2.17	1	2.50	2	2.33			0.87	0.03	22.40	0.920
Contact history	**Contact with someone positive**												
	No	23	51.11	13	34.21	36	43.37	83	95.4	Ref			
	Yes	22	48.89	25	65.79	47	56.63			2.01	0.83	4.98	0.124
Current or previous (last 3 months) COVID-19 symptoms	**Fever**												
	No	40	86.96	31	75.61	71	81.61	87	100.0	Ref			
	Yes	6	13.04	10	24.39	16	18.39			2.15	0.72	6.93	0.179
	**Difficulty breathing**												
	No	40	86.96	34	82.93	74	85.06	87	100.0	Ref			
	Yes	6	13.04	7	17.07	13	14.94			1.37	0.42	4.64	0.600
	**Tiredness**												
	No	33	71.74	32	78.05	65	74.71	87	100.0	Ref			
	Yes	13	28.26	9	21.95	22	25.29			0.71	0.26	1.89	0.500
	**Body pain**												
	No	40	86.96	26	63.41	66	75.86	87	100.0	Ref			
	Yes	6	13.04	15	36.59	21	24.14			3.85	1.38	11.98	**0.013**
	**Throat pain**												
	No	37	80.43	30	73.17	67	77.01	87	100.0	Ref			
	Yes	9	19.57	11	26.83	20	22.99			1.51	0.55	4.20	0.423
	**Diarrhea**												
	No	42	91.30	35	85.37	77	88.51	87	100.0	Ref			
	Yes	4	8.70	6	14.63	10	11.49			1.80	0.48	7.52	0.391
	**Chest pain**												
	No	42	91.30	36	87.80	78	89.66	87	100.0	Ref			
	Yes	4	8.70	5	12.20	9	10.34			1.46	0.36	6.28	0.594
	**Dry cough**												
	No	39	84.78	28	68.29	67	77.01	87	100.0	Ref			
	Yes	7	15.22	13	31.71	20	22.99			2.59	0.94	7.67	0.073
	**Loss of sense of smell or taste**												
	No	42	91.30	24	58.54	66	75.86	87	100.0	Ref			
	Yes	4	8.70	17	41.46	21	24.14			7.44	2.43	28.20	**0.001**
	**Headache**												
	No	30	65.22	27	65.85	57	65.52	87	100.0	Ref			
	Yes	16	34.78	14	34.15	30	34.48			0.97	0.40	2.36	0.950
Previous COVID-19 test	**Previous COVID-19 test**												
	No	35	76.09	36	87.80	71	81.61	87	100.0	Ref			
	Yes	11	23.91	5	12.20	16	18.39			0.44	0.13	1.35	0.166
Companion animals	**Companion animal**												
	No	24	53.33	21	52.50	45	52.94	85	97.7	Ref			
	Yes	21	46.67	19	47.50	40	47.06			1.03	0.44	2.44	0.939
	**Dog**												
	No	31	70.45	21	53.85	52	62.65	83	95.4	Ref			
	Yes	13	29.55	18	46.15	31	37.35			2.04	0.83	5.04	0.121
	**Cat**												
	No	34	77.27	35	89.74	69	83.13	83	95.4	Ref			
	Yes	10	22.73	4	10.26	14	16.87			0.39	0.11	1.36	0.139
	**Others**												
	No	42	95.45	39	100.00	81	97.59	83	95.4	Ref			
	Yes	2	4.55	0	0.00	2	2.41			0.22	0.01	4.62	0.326
Health history	**HIV**												
	No	45	97.83	40	97.56	85	97.70	87	100.0	Ref			
	Yes	1	2.17	1	2.44	2	2.30			1.13	0.04	29.07	0.934
	**Syphilis**												
	No	46	100.00	39	95.12	85	97.70	87	100.0	Ref			
	Yes	0	0.00	2	4.88	2	2.30			5.89	0.27	126.28	0.257
	**Hepatitis**												
	No	45	97.83	40	97.56	85	97.70	87	100.0	Ref			
	Yes	1	2.17	1	2.44	2	2.30			1.13	0.04	29.07	0.934
	**Cardiovascular disease**												
	No	36	78.26	29	70.73	65	74.71	87	100.0	Ref			
	Yes	10	21.74	12	29.27	22	25.29			1.49	0.56	4.00	0.421
	**Tuberculosis**												
	No	46	100.00	41	100.00	87	100.00	87	100.0	Ref			
	Yes	0	0.00	0	0.00	0	0.00			1.52	0.82	2.82	0.183
	**Diabetes**												
	No	43	93.48	38	92.68	81	93.10	87	100.0	Ref			
	Yes	3	6.52	3	7.32	6	6.90			1.13	0.20	6.43	0.884
	**Others**												
	No	44	95.65	40	97.56	84	96.55	87	100.0	Ref			
	Yes	2	4.35	1	2.44	3	3.45			0.55	0.02	5.95	0.631
Wash hands with water and soap	**Water and soap**												
	Yes	44	97.78	39	95.12	83	96.51	86	98.9	Ref			
	No	1	2.22	2	4.88	3	3.49			0.44	0.02	4.80	0.513

Ref = Category of reference

* Statistically significant variables

Ref = Category of reference

* Statistically significant variables

When considering the health histories of both populations, the homeless population was significantly more affected by syphilis and tuberculosis when compared to shelter workers (*p* = 0.0009 and *p* = 0.0002, respectively). Previous or current history of HIV infection, syphilis, hepatitis, and tuberculosis were declared by 6.5%, 15.1%, 8.5%, and 11.4% of the respondent individuals experiencing homelessness, respectively (Table 1). Cardiovascular disease and diabetes were reported by 29.1% and 9.4% of individuals who are homeless, respectively (Table 1). Overall, the proportions of shelter workers with previous or current infectious diseases were lower, with no reports of tuberculosis and equal percentages of HIV infection, syphilis, and hepatitis, amounting to 2.3% each (Table 2). However, no statistical differences were observed in the proportion of individuals who are homeless and shelter workers affected by HIV or hepatitis (*p* = 0.246 and *p* = 0.814, respectively). Likewise, cardiovascular disease and diabetes were reported by 25.3% and 6.9% of the shelter workers, respectively (Table 2), with no statistical difference from individuals who are homeless (*p* = 0.511 and *p* = 0.493, respectively).

The bivariate analysis for the presence of anti-SARS-CoV-2 IgG antibodies in individuals who are homeless showed statistically significant associations in this population (Table 1), where age (30 to 60 and >60 years old; *p* = 0.007 and *p* < 0.001, respectively) was associated with higher rates of IgG seropositivity, but tobacco use (*p* = 0.006), marijuana use (*p* = 0.003), and pet ownership (*p* = 0.008), including dogs (*p* = 0.044) and cats (*p* = 0.046) where associated with lower rates of IgG seropositivity (possible protective factors).

In the bivariate analysis of the shelter workers, the presence of anti-SARS-CoV-2 IgG antibodies was statistically associated with the city of origin (*p* = 0.025) and ethnicity (*p* = 0.015), where being from outside São Paulo and Black (compared to white) was associated with a higher seropositivity rate, as well as body pain (*p* = 0.013) and loss of sense of smell or taste (*p* = 0.001) as previous or current symptoms (possible risk factors).

In the multivariate analysis, a significantly increased risk of IgG seropositivity was observed in persons who are homeless of the age groups from 30 to 60 years old (OR 6.26, CI: 1.91–20.56) and older than 60 years old (OR 10.88, CI: 2.35–50.44) when compared to individuals up to 30 years old (Table 3). In other words, adults and elderly individuals who are homeless were 6.26 and 10.88 times more likely to be IgG seropositive than younger individuals (up to 30 years old), respectively. Finally, tobacco use (OR 0.37, CI: 0.17–0.81), body pain (OR 0.34, CI: 0.13–0.87) as current or past SARS-CoV-2 symptoms, and dog ownership (OR 0.22, CI: 0.07–0.24) were all detected as protective factors for SARS-CoV-2 exposure in persons who are homeless (Table 3).

**Table 3:** Final logistic model for analyzing risk factors associated with seropositivity of anti-SARS-CoV-2 IgG in 203 persons experiencing homelessness of São Paulo, Brazil.

	Adjusted OR	95% CI		p-value
		Lower	Higher	
Age (Ref = Less than 30 years old)*				
From 30 to 60 years old	6.26	1.91	20.56	**0.003**
More than 60 years old	10.88	2.35	50.44	**0.002**
**Gender (Ref = Male)**				
Female	0.96	0.25	3.76	0.959
Others	0.00	0.00	Inf.	0.996
**Race/Ethnicity (Ref = White)**				
Black	2.48	0.84	7.31	0.099
Mixed (Brown—Pardo)	1.33	0.54	3.29	0.537
Indigenous Brazilian	14.25	0.58	352.70	0.105
Schooling level (Ref = Higher education)*				
Elementary school	0.52	0.12	2.21	0.377
High school	0.24	0.06	0.96	0.043
**Assistance by the government healthcare program (Ref = Yes)**				
No	0.55	0.24	1.24	0.148
Tobacco (Ref = No)*				
Yes	0.37	0.17	0.81	**0.013**
**Injectables (Ref = No)**				
Yes	0.00	0.00	Inf.	0.990
**Fever (Ref = No)**				
Yes	2.48	0.92	6.63	0.071
Body pain (Ref = No)*				
Yes	0.34	0.13	0.87	**0.024**
**Loss of sense of smell or taste (Ref = No)**				
Yes	2.45	0.76	7.89	0.133
Dog owning (Ref = No)*				
Yes	0.22	0.07	0.74	**0.015**
**Cat owning (Yes x No)**				
Yes	0.22	0.03	1.49	0.122

Ref = Category of reference

* Statistically significant variables

There were no common risk or protective factors for IgG seropositivity between the homeless population and shelter workers. A higher risk for seropositivity was seen in Black shelter workers (OR 4.84, CI: 1.06–22.04) when compared to white shelter workers and in those that experienced loss of sense of smell or taste (OR 6.29, CI: 1.32–29.98) in the past months (Table 4).

**Table 4:** Final logistic model for the analysis of risk or protective factors associated with seropositivity of anti-SARS-CoV-2 IgG in 87 shelter workers of São Paulo, Brazil.

	Adjusted OR	95% CI		p-value
		Lower	Higher	
**Origin (Ref = São Paulo)**				
Others	2.66	0.73	9.63	0.136
**Age (Ref = Less than 30 years old)**				
From 30 to 60 years old	0.85	0.20	3.70	0.829
More than 60 years old	0.37	0.01	9.19	0.541
**Gender (Ref = Male)**				
Female	0.33	0.09	1.19	0.092
**Race/Ethnicity (Ref = White)** *				
Black	4.84	1.06	22.04	**0.041**
Mixed (Brown—Pardo)	2.51	0.54	11.77	0.243
**Body pain (Ref = No)**				
Yes	2.75	0.68	11.20	0.157
**Loss of sense of smell or taste (Ref = No)** *				
Yes	6.29	1.32	29.98	**0.021**

Ref = Category of reference

* Statistically significant variables

## Discussion

The study herein reports a high seroprevalence of SARS-CoV-2 infection in persons who are homeless and shelter workers from a large day-shelter in São Paulo city. The molecular results have ruled out active SARS-CoV-2 infection at the time of sampling and suggest that the high observed seroprevalence may be a consequence of the exposure to the first wave of SARS-CoV-2 in the city. With similar seroprevalence, both populations (homeless and shelter workers) were equally exposed to the virus, with a high probability that the daily agglomeration potentiated transmission at the shelter. The observed seroprevalence rates were significantly higher than the crude seroprevalence of 17.1% in blood donors from São Paulo observed a month later from this study (Sept 7^th^ to 29^th^, 2020) [19]. The prevalence was also higher than reported worldwide; in a systematic review comprising 23 countries, including Brazil, the SARS-CoV-2 seroprevalence in the general population varied from 0.4% (8/816) in Malaysia to 22.1% (117/528) in Iran as of August 2020 [20]. This finding reflects the current vulnerability individuals who are homeless and related shelter workers were subjected to, corroborating observations that the pandemic response has amplified and deepened current inequalities [2].

The seroprevalence of SARS-CoV-2 found in the homeless population also exceeds the crude seroprevalence rates reported in blood donors from the severely hit Amazon region from March to October 2020, which reported the highest prevalence in Brazil of 46.3% (422/911) in June 2020 [19]. A similarly high seroprevalence (~50%) was observed in a study comparing slums with no-slums households in Mumbai, India [21]. The present study corroborates with others, demonstrating that socioeconomic vulnerability, such as homelessness and living in slums, are risk factors for increased exposure to SARS-CoV-2 [20] and demonstrates the importance of serosurvey to policies and decision-making strategies and pandemic preparedness.

The shelter workers evaluated in this study included healthcare and social assistance professionals, cooking, and maintenance personal. It has been well known that healthcare professionals are at a greater risk of exposure to SARS-CoV-2 than the general population [22]. Strikingly, the seroprevalence of 47.1% (41/87) reported herein is higher than those of healthcare workers from other countries, ranging from 0 to 45.3% in a systematic review of 49 studies from North America, Asia, Europe, and Africa [23]. Notably, the prevalence rates vary according to the pandemic’s timing and location, rate of participation, type of healthcare worker, direct contact with patients, demographics, and socioeconomic conditions, among others [24]. Despite such variations, the closest seroprevalence rate to this study was 45.3% (87/200) in frontline health workers in the U.K. at the peak of the first wave of pandemics [25]. Different from our study, the overall prevalence in the U.K. study included cumulative results from baseline (25% seropositivity) and follow-up (19.0%) and RT-qPCR positive results (21%) results. Exposure to the community and access to PPE were important risk factors for virus exposure. In our study, hundreds of people visited the shelter daily, and the workers did not have access to appropriate PPE such as N95 or surgical masks; they used personal homemade masks. These factors likely contributed to the high SARS-CoV-2 seropositivity observed in the shelter workers herein.

Rates of seropositivity were expected to be higher than active infection (i.e., viral RNA detection via RT-qPCR) as antibodies remain present in serum after infection [26–28] and represent the cumulative exposure to SARS-CoV-2 in the population. Also, significantly lower O.D. values for IgG titers were observed in persons who are homeless compared to shelter workers. Possible reasons for such difference may include the time of infection, COVID-19 disease severity [29], and/or health conditions (e.g., malnutrition, comorbidities, substance abuse, etc.). We also cannot discount the possibility of false negatives in the ELISA assay due to low antibody titers and immunological window, which could mean that this population was even more affected by the pandemic than the percentage estimated herein. Thus, serologic testing plays a critical role in understanding the SARS-CoV-2 infection in different populations, and like in this study, helps identify segments of the population at a higher risk for infection.

Interestingly, active tobacco smoking status in individuals who are homeless was associated with a lower (protective) prevalence of SARS-CoV-2 antibodies. It is still controversial if tobacco smoking reduces or increases the risk of contracting SARS-CoV-2 and if smoking interferes with developing the more severe disease. There is documented evidence corroborating with the study herein, such as one study in Chicago shelters showing that individuals experiencing homelessness who were current smokers were less likely to be infected with SARS-CoV-2 [30]. Similarly, studies in China and across Europe showed a lower prevalence of hospitalized current smoker patients than the general population [31,32]. Another study in France showed the protective effect of smoking in non-hospitalized and hospitalized patients and reported a lower probability of developing symptomatic or severe disease [33]. Similar studies in China showed a lower prevalence of smokers in patients with poor outcomes [34] and a protective effect against severe diseases associated with past smoking [35]. Many researchers have hypothesized that the anti-inflammatory effects of nicotine [32,36] or the nicotinic receptors [37] could play a protective role in the pathophysiology of COVID-19; these hypotheses have not been proven to date.

On the other hand, it is necessary to emphasize that several studies demonstrate the harms of smoking concerning the progression, increased vulnerability to severe disease, and worse outcome of COVID-19 [38,39], as well as other respiratory disorders [40]. The most accepted explanation for the greater risk of severe disease is the increased ACE2 expression, the receptor implicated in virus-cell recognition in the bronchial epithelium [41–43]. This hypothesis was recently challenged by a study showing no significant mRNA expression levels of ACE2 receptors in individuals who never smoked compared with smokers [44]. Thus, such subject remains uncertain and beyond this study’s scope. Most importantly, the WHO has emphasized the well-established tobacco use risks and strongly recommends tobacco cessation [45].

An interesting finding of our study was the fact that dog ownership by individuals who are homeless was identified as a protective factor against SARS-CoV-2 infection. Most of the São Paulo city’s sleep-in shelters do not have adequate space to accommodate pets in their facilities. Thus, we speculate that persons who are homeless with dogs tend to agglomerate less than individuals without pets, justifying the protective factor. Also of interest was the fact that body pain was associated with lower seropositivity to SARS-CoV-2. Body pain is a symptom of SARS-CoV-2 infection but also of many other tropical diseases that are common in Brazil, such as dengue, zika, and chikungunya fevers, and leptospirosis, as well as behavioural characteristics, such as drug use or mental health illness, and poor housing conditions. Thus, other diseases or conditions not assessed herein may have acted as confounding factors to this association. As expected, loss of sense of smell or taste had significantly increased the risk of seropositivity in shelter workers, in agreement with 440/567 (77.6%) individuals during the COVID-19 pandemic peak in London, who have also presented SARS-CoV-2 anftibodies [46].

The homeless population herein showed complex health histories, particularly associated with infectious diseases (HIV, syphilis, and tuberculosis). However, previous history of STDs and tuberculosis were not found to be associated with SARS-CoV-2 infection. Despite growing concerns [47,48], it remains unknown whether people with previous history of tuberculosis, a respiratory disease, are more susceptible to SARS-CoV-2 infection or the development of severe COVID-19 [48]. In this study, we did not evaluate latent TB infection or TB and SARS-CoV-2 co-infection; and it may not be possible to rule out if people with previous TB history had worsened clinical symptoms.

Black shelter workers herein presented higher likelihood of being seropositive for SARS-CoV-2 than white shelter workers, as previously shown that Black people were at higher risk of contracting COVID-19 than white people [49]. In Brazil, Black individuals have been reportedly more likely to live in poverty than white individuals, along with less access to basic needs, including healthcare [50]. Poverty-associated household and public transportation overcrowding hinder individuals’ ability to protect themselves against SARS-CoV-2 infection [51]. These findings highlight the existence of social inequalities in health for which the role of structural racism should be further studied, as previously reported in the USA [52–55]. Finally, despite the robust outcome found herein, the relatively low homeless sampling does not exclude confounding factors such as center employment, jobs with involuntary gathering such as cooking and cleaning (as opposed to social and medical assistances), and workers living on households of same neighborhood, which may have existed at the time and could partially or entirely produce the observed association herein.

This study has limitations. Although the analyzed homeless population showed similar sociodemographic characteristics (i.e., origin, age, gender, race/ethnicity, level of education) to a recent survey in São Paulo city [56], all participants are from a single region of the city. Thus, the results of SARS-CoV-2 seroprevalence should not be extrapolated to the entire homeless population of São Paulo city. The lack of information about the proportion of individuals who are homeless of the Mooca subregion accessing this shelter at some point in time or daily also hampers our ability to extrapolate these results to the entire Mooca homeless population. Future studies should include randomized, larger sample size, and better geographic representation. We cannot exclude, for example, the possibility that agglomerations in this day-shelter may have increased populational exposure to SARS-CoV-2, and thus, the same conditions may not apply to individuals who are homeless or shelter workers in other regions of the city. Additionally, all participants made self-declarations to the questionnaire related to their own history and perception of illness and symptoms, which may have led to inaccurate reports due to forgetfulness or lack of knowledge or understanding.

## Conclusion

In conclusion, the present study reports a high SARS-CoV-2 IgG seroprevalence in individuals who are homeless and related shelter workers from a day-shelter in São Paulo, Brazil. At the time of the study (August 2020), both homeless and social worker populations showed no active SARS-CoV-2 infection, indicating that they were likely exposed sometime within the pandemic’s first peak in the city. The homeless population of São Paulo has been exponentially increasing over the past years, and current socioeconomic and housing programs are not enough to lift individuals out of the streets. Our study indicates that such living conditions led this homeless population to be severely affected by the pandemic. The effects of widespread infection were also not accounted for by official authorities, underscoring the importance of this study in providing the rationale needed to protect this population from the risk of infection by SARS-CoV-2 amid new surges of the virus. We advocate for the accountability of the number of cases and deaths among individuals experiencing homelessness, targeted vaccination of this population, healthcare programs to shelters, diagnostic testing, and further investment in housing, cash transfer, and employment programs to attend individuals in vulnerable situations such as homelessness in the city of São Paulo.

Our study has also shown significant risk and protective factors for SARS-CoV-2 infection, including that Black shelter workers were at higher risk of SARS-CoV-2 infection when compared with the white shelter workers. This finding indicates a difference in exposure according to race, providing evidence of race-associated health disparities for which the whole of structural racism should be further investigated.
